# Atlantic cod (*Gadus morhua*) embryos are highly sensitive to short-term 3,4-dichloroaniline exposure

**DOI:** 10.1016/j.toxrep.2021.10.006

**Published:** 2021-10-11

**Authors:** Bjørn Henrik Hansen, Julia Farkas, Stefania Piarulli, Silvia Vicario, Bjarne Kvæstad, David R. Williamson, Lisbet Sørensen, Emlyn John Davies, Trond Nordtug

**Affiliations:** a SINTEF Ocean, 7465, Trondheim, Norway; b University of Milano-Bicocca, Piazza della Scienza 1, Milan, Italy; c Centre for Autonomous Marine Operations and System (AMOS), Department of Marine Technology, Norwegian University of Science and Technology, NTNU, Norway

**Keywords:** 3,4-DCA, Emerging pollutants, Comparative ecotoxicity, Embryotoxicity, Cardiotoxicity

## Abstract

3,4-dichloroaniline (3,4-DCA) is one of the most widely produced anilines world-wide, used in plastic packaging, fabrics, pharmaceuticals, pesticides, dyes and paints as well as being a degradation product of several pesticides. 3,4-DCA has been detected in freshwater, brackish and marine environments. Although freshwater toxicity thresholds exist, very little toxicological information is available on marine and cold-water species. In this study, we exposed Atlantic cod (*Gadus morhua*) embryos (3–7 days post fertilization) to 3,4-DCA concentrations ranging from 8−747 μg/L for 4 days followed by a recovery period in clean sea water until 14 days post fertilization (dpf). The cod embryos were significantly more sensitive to acute 3,4-DCA exposure compared to other species tested and reported in the literature. At the highest concentration (747 μg/L), no embryos survived until hatch, and even at the lowest concentration (8 μg/L), a small, but significant increase in mortality was observed at 14 dpf. Delayed and concentration-dependent effects on surviving yolk-sac larvae, manifested as cardiac, developmental and morphometric alterations, more than a week after exposure suggest potential long-term effects of transient embryonic exposure to low concentrations of 3,4-DCA.

## Highlights

• Atlantic cod embryos were exposed to for 4 days to 8−747 μg 3,4-DCA/L followed by recovery in clean sea water for 7 days.
• Compared to comparable tests un literature, Atlantic cod was far more sensitive to acute exposure.
• Cardiac, developmental and morphometric alterations to larvae suggest long-term effects of transient embryonic exposure.

## Graphical abstract

**Figure:**
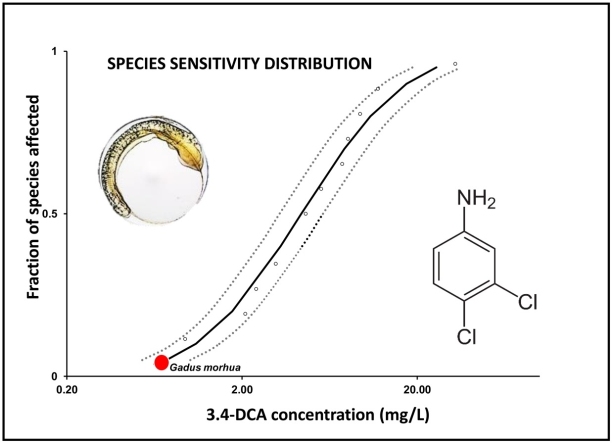

## 1 Introduction

3,4-dichloroaniline (3,4-DCA) is one of the most widely produced and used aniline derivates in the EU, USA and Asia [1,2]. 3,4-DCA is a precursor and intermediate product in the chemical synthesis of several industrial products, including herbicides, dyes and paints [3,4]. It is, for example, an intermediate of 3,4-dichlorophenylisocynate, used for the production of phytosanitary products such as propanil, linuron, diuron and neburon [1]. Microbial degradation of these pesticides may also generate 3,4-DCA, which can be more toxic and persistent in the environment than its parent compounds e.g., propanil [[5], [6], [7]]. 3,4-DCA is further used for synthesising azo dyes used in plastic packaging, fabrics and pharmaceuticals [8,9].

Chloroanilines are relatively mobile chemicals, known to easily diffuse into the natural environment and are generally difficult to remediate [10]. As a result of intense production and use, 3,4-DCA is released via waste waters into freshwater channels and rivers and can be transported to the marine environment [[11], [12], [13]]. Due to its tendency to form covalent bonds with the organic fraction of sediments and suspended matter, 3,4-DCA is subjected to vertical transport from the water column to sediments and therefore, constituting a potential risk for marine benthopelagic species [14].

3,4-DCA has been detected in various fresh-, brackish- and marine aquatic environments at concentrations up to 0.7 μg/L [[15], [16], [17], [18], [19], [20]]. As part of the NORMAN list (http://www.norman-network.net/), it is widely recognized as an emerging aquatic pollutant and has a well-documented toxicity towards many standard test species, including several freshwater fish during their early life stages (ELS) [21,22]. 3,4-DCA is thus used as positive control substance in the Fish Embryo Acute Toxicity (FET) test, with a target LC_50_ concentration of 4000 μg/L as test validity criterium [23]. Concentrations causing acute toxicity in ELS of other freshwater fish species vary, with reported 96h-LC_50_ values ranging between 940 μg/L in Arabian killifish (*Aphanius dispar*) and 33 000 μg/L in Javanese medaka (*Oryzias javanicus*) [24,25]. Generally, acute toxicity of 3,4-DCA is described as relatively low (96h-LC_50_>1000 μg/L for most species) compared to its chronic toxicity, with acute to chronic ratios (ARC) in fish reaching from approximately 20 (guppy; *Poecilia reticulata*) to 1000 (fathead minnow; *Pimemphales promelas*) (for review see Crossland)[26].

Applying QSAR modelling, polar narcosis was identified as general toxic mode of action of 3,4-DCA [27]. Recent molecular studies suggest that 3,4-DCA exposure causes interference with cell cycle control, biotransformation and metabolic processes [22,28]. Several studies further reported endocrinological impacts, including changes in gene expression along the hypothalamic-pituitary-gonadal (HPG) axis and alterations of 17β-estradiol and testosterone plasma concentrations in both sexes of zebrafish (*Danio rerio*) and Nile tilapia (*Oreochromis niloticus*) [[29], [30], [31]]. Ibrahim and co-authors [65] (2021) further documented reduced spawning rates and lowered gonadosomatic indices (GSI) in female Javanese medaka (*O. javanicus*), and 3,4-DCA exposure has also been shown to cause delayed development, altered heart rates, pericardial and yolk sac edema and various skeletal deformations fish embryos and larvae [24,32].

While acute toxicity and sublethal effects are relatively well studied in freshwater species, knowledge of impacts on marine fish, especially cold-water fish, is scarce. This is concerning as studies have shown that sub-Arctic and Arctic species may be more susceptible to toxicants than those from temperate regions [[33], [34], [35]]. Atlantic cod (*Gadus morhua*) is an important benthopelagic species in the Northern Atlantic Ocean from an ecological, commercial and cultural perspective, being of great significance for fisheries in Northern countries, such as Norway [36,37]. Due to its key ecological role, assessment of its sensitivity remains a critical issue [38]. Further, 3,4-DCA and its precursors have been listed as “River basin specific pollutants of priority importance” by the River Basin Management Plan (RBMP) for the implementation of the Water Framework Directive to effectively manage and protect aquatic resources. It is thus important to assess the impact on cod, particularly on its sensitive early life stages.

The main aim of the study presented here was to investigate developmental effects and determine toxicity threshold levels of 3,4-DCA exposure on cod (*Gadus morhua*) ELS. We applied a standardized experimental protocol developed for this species to determine acute toxicity (reduction in embryonic survival and hatching) and cardiotoxic, morphological and developmental biomarkers in larvae following short-term embryonic exposure.

## 2 Materials and methods

### 2.1 Chemical and generation of exposure solutions

3,4-Dichloroaniline was purchased from Sigma-Aldrich (purity > 98 %). Natural sea water was initially collected through a sand filter from 70 m depth in the Trondheimsfjorden. To produce a stock solution, 3000 μg 3,4-DCA was added to 1 L of filtered (1 μm, Sterivex) sea water and stirred vigorously for 2 h at 20 °C. The solution was then left to equilibrate for 30 min before being diluted to 3 L filtered seawater to reach a nominal concentration of 1000 μg/L, which was used as the highest exposure concentration in the test. This solution was further diluted with filtered seawater to produce the other exposure solutions at nominally: 25, 50, 150, 250, and 500 and 1000 μg/L.

### 2.2 Chemical analyses

Water samples from the whole dilution series were analyzed for 3,4-DCA concentrations to verify exposure concentrations. Water samples (approximately 800 mL) were taken from each dilution, acidified with HCl (15 % aq. solution, Sigma-Merck) to reach pH < 2, and extracted using dichloromethane (DCM, analytical grade, purity verified in-house, Rathburn). Phenanthrene-*d*10 was used as surrogate internal standard to account for target analyte losses during the extraction step. The extract was then dried over anhydrous sodium sulphate and concentrated by gentle solvent evaporation. Finally, fluorene-*d*10 was added as internal standard to determine recovery rates.

Analysis of extracts was performed using an Agilent 7890B GC coupled to an Agilent 5977A MSD. Samples (1 μL) were introduced at 325 °C in pulsed splitless mode. Separation was achieved using a Zebron ZB-5MSplus column (60 m length, 0.25 μm film thickness and 0.25 mm internal diameter). The carrier gas was helium 6.0 at a constant flow of 1.0 mL/min. The column oven temperature was programmed at 40 °C (1.4 min), ramped by 6 °C/min until 220 °C and 4 °C/min until 325 °C (10 min hold). The transfer line temperature was 325 °C, the ion source temperature 230 °C and the quadrupole temperature 150 °C. The ion source was operated in fullscan mode (50−300 *m/z*) with a solvent delay of 12 min. Quantification of 3,4-DCA was done by *m/z* 163 using an average response factor of a 4-level calibration curve (5−100 μg/mL) after normalization to the internal standard (fluorene-*d*10, *m/z* 176). Using phenanthrene-d10 as a surrogate internal standard to account for target analyte losses during the extraction step provided a recovery of 93 %.

The concentrations measured in exposures were somewhat lower than the targeted nominal concentrations of 25−1000 μg/L, being 8, 27, 108, 220, 343 and 747 μg/L. This was probably due to incomplete dissolution of 3,4-DCA when preparing the stock solution. All toxicological data referred to hereafter are related to the measured concentrations.

### 2.3 Acquisition of Atlantic cod eggs

Fertilized cod (*Gadus morhua*) eggs were supplied by NOFIMA. The eggs were obtained by strip-spawning one ovulating female from a broodstock at Havbruksstasjonen in Tromsø on April 1st and fertilized with milt from one male cod. Eggs were incubated over night at 4 °C and fertilization success determined (>75 %) the following morning. The eggs were shipped in 10 L of oxygenated sea water inside a Styrofoam box containing ice. Upon arrival, temperature (4 °C) and oxygen concentration (>8 mg/L) was measured. The eggs were then slowly acclimated to a temperature of 8.5 °C over the next 36 h before being used for toxicity testing.

### 2.4 Exposure and effects on hatching and survival

The test procedure used in this study was adapted from OECD Test No. 236: Fish Embryo Acute Toxicity (FET) using zebrafish (*Danio rerio*) as test species [23]. Atlantic cod eggs are comparable to zebrafish eggs in size, but in contrast, cod eggs have a lower density (pelagic eggs) and develop at lower temperature and over a longer period from fertilization to hatch (80–120 degree days, d°). Compared to the OECD guideline, we used larger exposure beakers (100 mL), an increased number of individuals (approximately 100 eggs per beaker) and a lower temperature (8.5 ± 1 °C).

Three days post fertilization (3 dpf) approximately 100 cod eggs were transferred to individual borosilicate beakers containing filtered sea water (controls; N = 6 replicates) or exposure solutions (100 mL; N = 3 replicates per concentration). The eggs were exposed for 96 h, and solutions were renewed every 24 h. After 96 h exposure, all eggs were transferred to new beakers containing filtered sea water for recovery. Upon hatching, larvae from replicate treatments were pooled into one larger beaker (500 mL). Mortality (determined as eggs displaying coagulated embryos on the bottom of the beaker) and hatching were monitored daily until 14 dpf. A complete timeline for the experiment is given in the Supporting Information (SI1, Table S1.1).

### 2.5 Microscopy, heart rate and morphometry

**Fig. 1:**
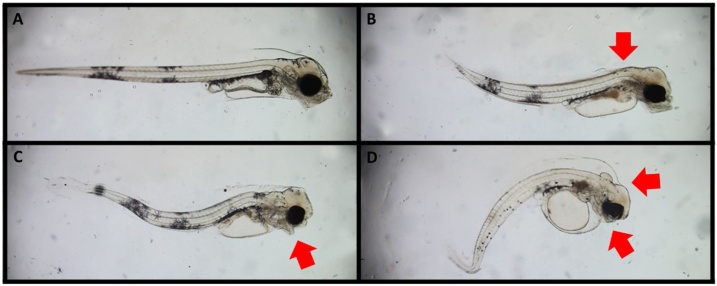
Images of Atlantic cod larvae (15 dpf). A: Normal larvae. B-D: Cod larvae after 4 days' embryonic exposure to 3,4-dichloroaniline. B: Larvae with severe lack of finfold inflation (indicated by arrow) and moderate spinal deformation, moderate craniofacial and jaw deformations. C: Larvae with moderate spinal deformations, severe jaw (indicated by arrow) and craniofacial deformations D: Larvae with severe craniofacial deformations and complete lack of jaw structures (indicated by arrows), severe spinal deformations and moderate fin fold deformations. All deformed larvae (B-D) also display larger yolk sizes compared to the normal larvae (A).

Effects on heart rate and morphometry were determined from images collected from 10 to 20 embryos (9 dpf) from one replicate of each treatment and 8–40 individual larvae (15 dpf) from each pooled replicate (except for the highest treatment where no larvae hatched). Images and videos were taken with a microscope (Eclipse 80i, Nikon Inc., Japan) equipped with Nikon PlanApo objectives (2x and 4x for egg videos and whole larvae images and 10x for close-up larvae images and videos), a 0.5x video adaptor and a CMOS camera (MC170HD, Leica Microsystems, Germany). Videos were used as a basis for heart rate (HR) analyses and determination of ventricle constriction (incidence of silent ventricle) in individual embryos/larvae using manual analyses. Larvae images were used for morphometric analyses (standard length, body area, yolk sac area and eye area) using automated analyses (AUTOMOMI) [66]. Ranking of craniofacial, jaw and spinal deformations as well as abnormalities in finfold inflation was performed by blinded image analyses as described previously [[39], [40], [41]]. Briefly, larvae were ranked as normal (severity degree 1), moderately deformed (severity degree 2) and severely deformed (severity degree 3). Examples of different degrees of deformations are given in Fig. 1.

### 2.6 Data base toxicity data on 3,4-DCA and species sensitivity distributions

To compare the sensitivity of cod ELS with other species, data were retrieved from the ECETOC database (https://cfpub.epa.gov/ecotox/search.cfm) and literature. The search included the CAS number (95,761), the keywords: fish, mortality, and test duration: 4 days. Only 3,4-DCA as chemical was included. No discrimination regarding organism age, exposure type, media type or test location was used. The data obtained can be found in Supporting Information (SI2, Table S2.1). Comparative toxicity was assessed using species sensitivity distributions (SSD) [42] and to prepare species sensitivity distribution plots, the CADDIS SSD Generator V1 macro for Microsoft Excel, available on the US-EPA website (https://www.epa.gov/caddis-vol4/caddis-volume-4-data-analysis-download-software), was applied. Where several LC_50_ values were available for the same species, average values were used.

### 2.7 Statistical analyses

Statistical analyses were conducted with GraphPad Prism V6.00 (GraphPad Software, Inc., CA, USA). Comparisons between treatments were performed with one-way ANOVA, followed by Tukey's multiple comparisons test or Kruskal-Wallis test, followed by Dunn's multiple comparison test for non-normal distributed data sets according to D'Agostino & Pearson omnibus normality test. Significance level was set to p < 0.05 unless otherwise stated. A nonlinear curve fit (third-order polynomial) was applied in figures displaying measured parameters plotted as a function of exposure concentrations.

## 3 Results and discussion

### 3.1 Acute toxicity and species sensitivity distribution

**Fig. 2:**
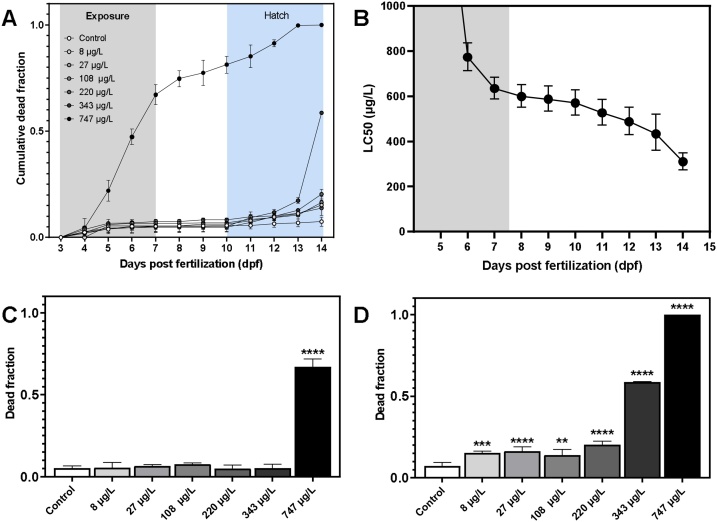
A: Cumulative dead fraction as a function of time (days post fertilization, dpf) for cod embryos exposed to 6 concentrations of 3,4-dichloroaniline between 3–7 dpf (indicated in grey area). Light blue area indicates hatching days. B: Estimated LC_50_ values (and confidence intervals) plotted as a function of time. Grey area indicates the exposure period. C: Cumulative dead fraction at the end of the exposure (7 dpf). D: Cumulative dead fraction at the end of the experiment (14 dpf). Data are given as average ± standard deviation (N = 6 for control, N = 3 for all 3,4-DCA exposures). Significantly higher dead fraction in 3,4 -DCA treatments than controls are given as **p < 0.01, ***p < 0.001 and ****p < 0.0001. (For interpretation of the references to colour in this figure legend, the reader is referred to the web version of this article).

Mortality was assessed from exposure start (3 dpf), throughout the exposure period (3–7 dpf) and to the end of the recovery period (14 dpf) (Fig. 2). The LC_50_ concentration after 96 h of exposure (7 dpf) was 635.1 μg/L (95 % CI: 589.0–684.8 μg/L), and at the highest exposure concentration, approximately 70 % of the embryos had died by the end of exposure (Fig. 2C). There was, however, a lack of clear dose-response for mortality at the end of exposure, and lack of linearity has been previously observed in fish embryos exposed to pollutants [43]. At the end of the recovery period (14 dpf), however, all 3,4-DCA-exposed groups displayed significantly higher cumulative mortality than controls (Fig. 2 D) and in a more concentration-dependent manner, and the LC_50_ was estimated at 310.3 μg/L (95 % CI: 275.0–350.0). Estimated LC_50_ thresholds over time are shown in Fig. 2B. Accumulation and toxicity of organic pollutants is exposure dose- and duration-dependent but also life stage dependent [44]. The observed increase in mortality over time during recovery is caused by 3,4-DCA-induced maldevelopment during embryogenesis.

**Fig. 3:**
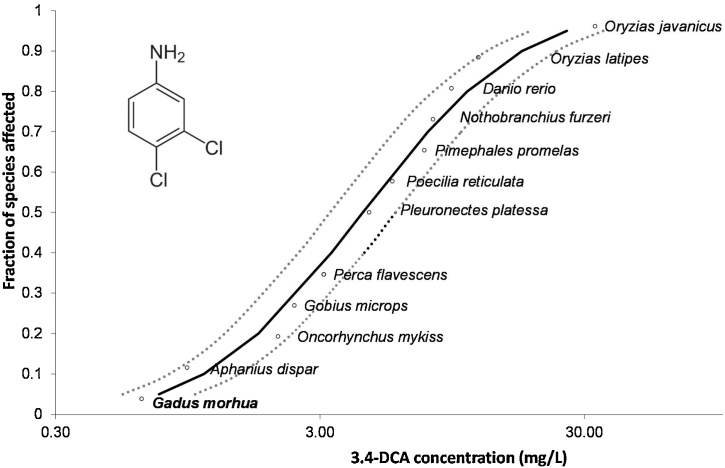
Species sensitivity distributions (SSD) based on fish 96h- LC_50_ data for 3,4-DCA (mg/L) obtained from the literature and this study (Gadus morhua).

Based on all ECETOC entries for 3,4-DCA LC_50_ values for fish, only the three-spined stickleback (*Gasterosteus aculeatus*) displayed LC_50_ than Atlantic cod embryos (360 μg/L). This was from an 26-day outdoor artificial stream study [45] and is not directly comparable to our short-term study. Comparable acute toxicity data from 13 different species (Supporting Information SI2, Table S2.1) were gathered from the literature and the ECETOC database. These included the five freshwater species turquoise killifish (*Nothobranchius furzeri*), yellow perch (*Perca flavescens*), European perch (*Perca fluviatilis*), fathead minnow (*P. promelas*) and zebrafish (*D. rerio*), the anadromous rainbow trout (*Oncohynchus mykiss*), the four species guppy (*P. reticulata*), common goby (*Gobius microps*), medaka (*Oryzias latipes*) and Javanese medaka (*O. javanicus*) found in both freshwater and brackish water and the two marine species European plaice (*Pleuronectes platessa*) and Arabian killifish (*A. dispar*). Compared to these acute toxicity data, Atlantic cod was the most sensitive fish species tested for 96 h acute toxicity with 3,4-DCA as shown in species sensitivity distribution (Fig. 3). Adema and Vink [46] tested 3,4-DCA on several different freshwater, euryhaline and marine fish species. Freshwater guppies were less sensitive than the marine guppies to exposure and displayed LC_50_ thresholds in the range 950–9000 μg/L. The euryhaline goby displayed an LC_50_ of 2400 μg/L when tested in sea water. The marine fish plaice (*P. platessa*) was less sensitive with an LC_50_ of 4600 μg/L. For the anadromous rainbow trout (*O. mykiss*) Hodson [47] reported an LC_50_ concentration of 1940 μg/L. With an LC_50_ of 32 870 μg/L, newly fertilized estuary-residing Javanese medaka (*O. javanicus*) embryos were the least sensitive (this database query). Comparable 96h- LC_50_ thresholds to Atlantic cod were documented for Arabian killifish (*A. dispar*), a ubiquitous species in the Arabian Gulf coastal water, which was tested at two different embryonic developmental stages by Saeed et al. [25]. The 50 % effect concentration (EC_50_), based on hatching success after 240 h exposure, was 480 μg/L and 1780 μg/L 3,4-DCA for embryos exposed before 6 h post fertilization (hpf) and after 168 hpf, respectively. This suggests that timing of exposure during embryogenesis is important when establishing toxicity thresholds. Temperature may also affect thresholds, as observed in killifish, where 96h- LC_50_ were 9750 μg/L at 24 °C and 6610 μg/L at 28 °C [48].

Short-term acute toxicity tests usually cover 24–96 h of exposure, assessing survival as a function of exposure concentration, but they reveal no information regarding delayed and/or sub-lethal toxicity. Our experiments followed hatching and survival for an additional week after exposure. The embryos started hatching on day 10 post fertilization, and the hatching period was considered ended at 14 dpf. Apart from the group exposed to 108 μg/L, all groups displayed earlier hatch compared to controls (p < 0.05), however, the differences from controls were less than one day (SI4, Figure S4.1). Early hatching was previously described as an escape strategy in fish under unfavourable conditions [49,50] and has previously been shown in Atlantic cod after exposure to produced water [39] and mine tailings [51]. Premature hatching was clearly associated with higher larvae mortality in the study by Farkas et al. [51], however, compared to our experiment, the premature hatching caused by mine tailing exposure occurred much earlier than controls (4 days prior to controls).

At day 14 post fertilization all remaining unhatched eggs were considered dead, and for the highest concentration no hatching was observed. Cumulative mortality (as fraction of total) is shown in Fig. 2A. Using a sigmoidal dose-response (variable slope), LC_50_ thresholds were estimated based on daily measurements of mortality (Fig. 2B) resulting in an LC_50_ concentration of 310.3 μg/L (CI: 275.0–350.0) at 14 dpf, in comparison to 635.1 μg/L directly after 96 h of exposure. This shows that delayed mortality occurs and should be considered when assessing chemical toxicity. Our findings agree with a previous study, reporting delayed mortality in 3,4-DCA exposed Javanese medaka (*O. javanicus*) [24]. After an exposure to 5000 μg/L for 96 h, almost no mortality was observed, but by 18 days post exposure all individuals had died. Similarly, a significant delayed mortality (96.7 %) was observed after recovering from the short-term exposure to 2500 μg/L 3,4-DCA [24].

### 3.2 Embryonic development

**Fig. 4:**
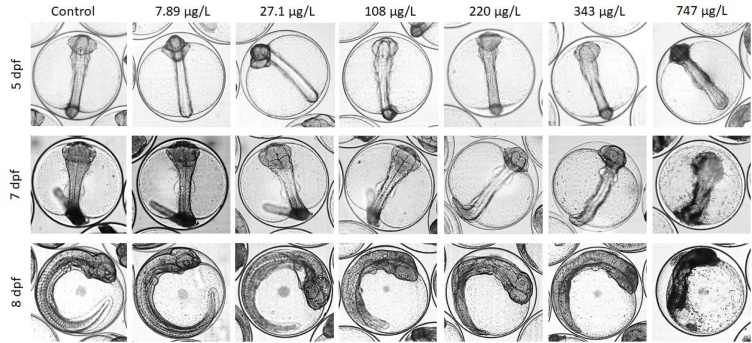
Images of representative embryos exposed to 3,4-dichloroaniline between 3 to 7 days post fertilization from all treatments 2 days into exposure (5 dpf), at the end of exposure (7 dpf) and one day after exposure (8 dpf). No scale bar is given, but the diameter of the eggs is approximately 1.3 mm.

At the onset of exposure embryos were in late developmental stage II, according to embryonic classification of cod [52], and at the end of the exposure period (7 dpf) the embryos of all treatments were at the end of stage III (segmentation stage) (Fig. 4). Embryos exposed to the highest concentration, however, showed signs of necrosis in the head region after 2 days of exposure (5 dpf) and were almost disintegrated by the end of exposure (7 dpf). This was even more pronounced one day after exposure (8 dpf). A concentration-dependent reduction in embryo length was observed (8 dpf). For controls and the two lowest concentrations the head almost reached the tip of the tail, whereas for the remaining treatments the distance between the tip of the tail and the head was longer (Fig. 4). This was not verified statistically due to difficulties in keeping a significant number of eggs oriented in identical manner to make reliable measurements.

### 3.3 Cardiac activity in embryos and larvae

**Fig. 5:**
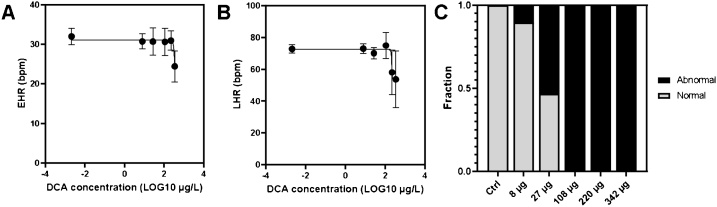
Cardiac activity. Heart rate of Atlantic cod embryos (EHR, 8 dpf, A) and larvae (LHR, 15 dpf, B) after 4 days' exposure (3–7 dpf) to 3,4-dichloroaniline plotted as a function of exposure concentration. Data are given as average ± standard deviation (N = 12–18 for eggs and N = 10–30). A non-linear fit slope (four parameters) is given (R^2^ = 0.41 for embryos and R^2^ = 0.39 for larvae). C: Fraction of larvae displaying normal (grey) and abnormal (black) ventricle constriction (silent ventricle).

One day after exposure (8 dpf), control embryos had an average heart rate of 32 ± 2 beats per minute (bpm). A significantly (p < 0.001) reduction in heart rate (24 ± 4 bpm) was recorded in the second highest treatment (342 μg/L), and none of the embryos at the highest concentration (746 μg/L), displayed heart activity. For concentration-dependent response, see Fig. 5A. Embryos exposed to the highest concentration did not hatch, so no heart rate measurements could be done on larvae from this group. (Fig. 5B). This is in agreement with observations of Ibrahim et al. [24] in Javanese medaka (*O. javanicus*) embryos exposed to 3,4-DCA from fertilization until 96 hpf to 5000 μg/L where at 13 days post exposure none of the medaka displayed beating hearts, and individuals in this group did not hatch.

As was observed for embryos, HR was reduced in cod larvae that were exposed to 3,4-DCA as embryos, with heartrates significantly different compared to controls (p < 0.001) in groups exposed to 220 and 343 μg/L (Fig. 5B). Larvae were also assessed for ventricular constriction, showing a concentration-dependent effect as the fraction of larvae with a silent ventricle increased with exposure concentration (Fig. 5C). Even at the lowest exposure concentration (8 μg/L), 10 % of the larvae had a silent ventricle. Over half of the larvae assessed displayed a silent ventricle after exposure to 27 μg/L as embryos, and none of the larvae presented ventricular constriction at any of the higher concentrations. Bradycardia and lack of ventricular constriction induced by chemical stressors is often associated with pericardial edema [53]. Interestingly, none of the cod larvae exposed to 3,4-DCA showed signs of a pericardial edema. In contrast, Javanese medaka (*O. javanicus*) displayed pericardial edema after exposure to 500 μg/L 3,4-DCA [24], and in fathead minnow (*P. promelas*) pericardial edema was observed after exposure to 700 μg/L [54].

### 3.4 Larvae morphometry and deformations

Results from the automated image analyses of larval standard length, body area, yolk area, eye diameter and eye-to-front distance, are shown in Table 1. Measurements were not possible for larvae exposed to the highest concentration because no larvae hatched from this treatment.

**Table 1:** Morphometric measurements of cod larvae (15 dpf) exposed to 3,4-dichloroaniline during embryogenesis (3–7 dpf). Data are given as average ± standard deviation (N = 8–28). Significant differences between exposed and control are given as*p < 0.05, **p < 0.01, ***p < 0.001 and ****p < 0.0001. NA = not analyzed (all dead).

Treatment	Ctrl	8 μg/L	27 μg/L	108 μg/L	220 μg/L	343 μg/L	747 μg/L
Standard length (mm)	4.8 ± 0.1	4.7 ± 0.1	4.5 ± 0.2**	4.3 ± 0.3****	3.8 ± 0.4****	3.4 ± 0.4****	NA
Body area (mm^2^)	1.42 ± 0.04	1.40 ± 0.04	1.35 ± 0.06**	1.30 ± 0.07****	1.18 ± 0.10****	1.22 ± 0.11****	NA
Yolk area (mm^2^)	0.08 ± 0.02	0.09 ± 0.02	0.11 ± 0.02**	0.13 ± 0.02****	0.16 ± 0.03****	0.23 ± 0.08****	NA
Eye diameter (μm)	284 ± 12	276 ± 10	273 ± 12	268 ± 10**	246 ± 19****	219 ± 17****	NA
Eye-to-front distance (μm)	86 ± 19	83 ± 17	79 ± 17	86 ± 19	62 ± 26**	44 ± 27***	NA

A significant reduction in standard length and body area was observed in a concentration-dependent manner, being significant for all exposure concentrations above the lowest. Our results align with previous studies on fathead minnow (*P. promelas*), which showed a reduced larval size after exposure to 3,4-DCA at concentrations as low as 7.1 μg/L [5]. Rare minnow (*Gobiocypris rarus*) displayed reduced larvae length after exposure to 2900 μg/L 3,4-DCA [55] and a concentration dependent reduction in standard length and body area was observed. An explanation for the reduced larvae size is likely due to a lower absorption of the yolk for growth. Significantly larger yolk sacs were observed in larvae from all treatments except the lowest 3,4-DCA treatment, and this was also observed in a concentration-dependent manner. Poor yolk absorption in combination with small larvae following embryonic exposure to crude oil has also been shown in Atlantic haddock (*Melanogrammus aeglefinus*), where mechanistic links to altered cholesterol synthesis and homeostasis was established using transcriptomics [56]. Proteomics analyses on zebrafish embryos exposed to 3,4-DCA revealed alterations in lipid-related pathways which are involved in mobilization of lipids from yolk sac during early larvae development, with two apolipoproteins (Apo1a and Apo1b) down-regulated by 3,4-DCA-exposure [22]. Utilization of the yolk sac through transport of lipids are pivotal processes for growth during the yolk-sac period, and disruption of such processes is a possible cause of the 3,4-DCA-treated cod larvae being smaller.

Morphometric analyses further showed that larvae exposed to 3,4-DCA as embryos had smaller eyes compared to control individuals (Table 1). The reduction is eye size was concentration dependent and significant for all groups, except the group exposed to the lowest concentration. This may be related to impacts on retinoids caused by 3,4-DCA exposure. Glyphosate-based herbicides displayed impacts on retinoids in zebrafish, including retinol, which is important for eye development and vision. In cod, retinol dehydrogenase and retinoid binding proteins were modulated by crude oil exposure [57], which has been mechanistically linked to the disruption of eye developmental processes causing a reduced eye size and a protruding eye lens [58]. Also, the distance between eye and forehead, previously used as an indication of craniofacial deformations in oil-exposed cod larvae [39,59], was shorter in larvae for the two highest exposure concentrations compared to controls (Table 1).

**Fig. 6:**
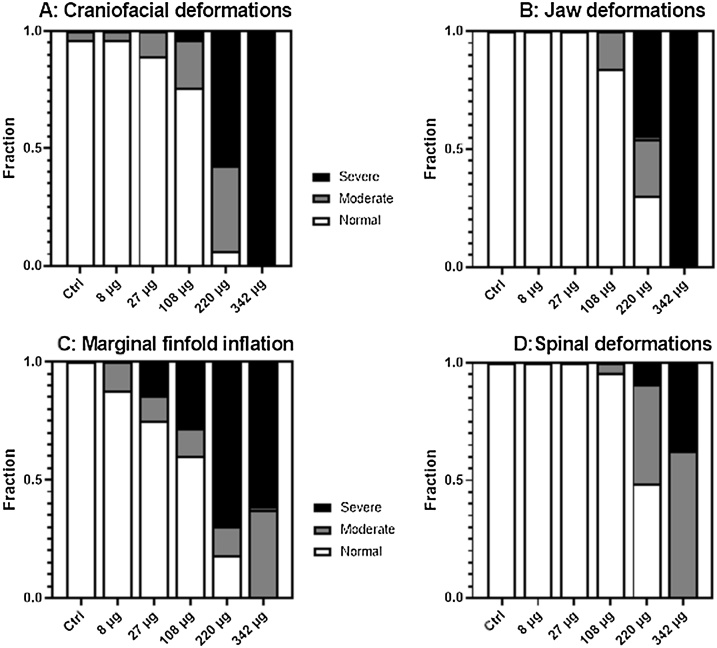
Fraction of Atlantic cod (15 dpf) larvae displaying deformations after 4 days’ embryonic exposure to 3,4-dichloroaniline at different concentrations. A: Craniofacial deformations. B: Jaw deformations. C: Lack of inflation of marginal finfold and D: Spinal deformations.

Larvae deformations were observed particularly at the highest concentrations (108−342 μg/L), and Fig. 6 shows the fraction of larvae with different deformations after being exposed to 3,4-DCA during embryogenesis. The incidences of all deformations were observed in concentration-dependent manners. Larvae exposed to 220 and 343 μg/L had craniofacial, jaw and spine deformations, and in the severe cases (Fig. 1C and D) jaws were almost not visible. Similar deformation phenotypes have been observed in Atlantic cod larvae exposed to crude oil [59,60], produced water effluents [39] and polycyclic aromatic hydrocarbons [40]. Statistical analyses showed that severe reduction of marginal finfold was the most sensitive deformation endpoint, as a higher frequency of larvae where this deformation was observed in exposures down to 27 μg/L. Lack of inflation of the marginal fin fold is shown in Fig. 1B, where the finfold is not separated from the cranium and/or spine. Being developed before hatch, the finfold is filled with extracellular matrix and is, in normal larvae, a continuous structure surrounding the larvae. The outer epidermis is separated from the brain, main body axis and internal organs by a subdermal space. This subdermal space consists of specialized cells regulating ion and water balance to maintain larval buoyancy ([61,56,62]) and facilitate larval swimming [63].

Short-term embryonic exposures that cause developmental and morphological alterations in larvae as observed in the present study may have long-lasting effects on fish development and survival probability. This was shown for polar cod (*Boregadus saida*) by Laurel et al. [64] where short-term embryonic exposure to low levels of crude oil caused similar effects as observed in the present work, including smaller larvae, larger yolk-sacs, deformations and cardiotoxicity. Polar cod larvae displaying severe deformations died before reaching 43 dph, possibly due to impaired swimming and foraging abilities. Even yolk-sac larvae that appeared morphologically normal but were somewhat smaller and with yolk sac alterations at hatch, displayed poor survival, altered lipid content and decreased growth to the end of their experiment (193 dpf) [64]. Thus, we argue that including the sub-lethal effects observed in larvae is relevant for assessing potential long-term effects of embryonic exposure in Atlantic cod.

## 4 Conclusions

This study of Atlantic cod embryos demonstrates that the early life stage of this cold-water marine fish species is more sensitive to 3,4-DCA than freshwater species previously studied. Cardiac and morphometric responses clearly show that these endpoints are important to properly assess toxicity to early life stages. By including these sub-lethal endpoints, predictions of more long-term effects may be obtained, as shown by Laurel et al. [64]. Concentration-dependent responses were observed for most of the effect parameters studied, and significant effects were observed for all but the lowest concentration, providing evidence for a no-effect concentration (NOEC) for Atlantic cod of 8 μg/L. This threshold is in fact in the range of 3,4-DCA concentrations measured in rivers and estuarine waters [15,20]. Importantly, chemical validation of exposure solutions also showed that nominal values for 3,4-DCA toxicity experiments may be inaccurate. This suggests that several reported values in the literature may underestimate 3,4-DCA toxicity. Furthermore, as shown in the literature, different developmental stages and temperatures may affect toxicity thresholds. As developmental rate of fish embryos is highly temperature-dependent, shorter exposure period than the four days utilized in the present study may cause similar toxicity thresholds at higher temperatures.

## CRediT authorship contribution statement

**Bjørn Henrik Hansen:** Conceptualization, Methodology, Formal analysis, Investigation, Data curation, Writing - original draft, Writing - review & editing, Visualization, Project administration, Funding acquisition. **Julia Farkas:** Formal analysis, Investigation, Writing - original draft, Writing - review & editing. **Stefania Piarulli:** Writing - original draft, Writing - review & editing. **Silvia Vicario:** Formal analysis. **Bjarne Kvæstad:** Methodology, Formal analysis. **David R. Williamson:** Data curation, Writing - review & editing. **Lisbet Sørensen:** Formal analysis, Investigation, Methodology, Writing - original draft. **Emlyn John Davies:** Data curation, Writing - review & editing. **Trond Nordtug:** Data curation, Writing - review & editing.

## Declaration of Competing Interest

The authors declare that they have no known competing financial interests or personal relationships that could have appeared to influence the work reported in this paper.
