# Supplementary material for: Atlantic cod (Gadus morhua) embryos are highly sensitive to short-term 3,4-dichloroaniline exposure
Source: Toxicol Rep. 2021 Oct 11;8:1754–61. doi: 10.1016/j.toxrep.2021.10.006 (PMC8523877; doi:10.1016/j.toxrep.2021.10.006)
Supplement: Supplementary file 1 [file mmc1.pdf]

SUPPORTING INFORMATION

Atlantic cod (*Gadus morhua*) embryos are highly sensitive to short-term 3,4-dichloroaniline exposure

Bjørn Henrik Hansen[1*], Julia Farkas[1], Stefania Piarulli[1], Silvia Vicario[3], Bjarne Kvæstad[1], David R. Williamson[1], Lisbet Sørensen[1] & Trond Nordtug[1]

[1]SINTEF Ocean, 7465 Trondheim, Norway

[2]University of Milano-Bicocca, Piazza della Scienza 1, Milan, Italy

*Corresponding author: Bjørn Henrik Hansen. E-mail: bjorn.h.hansen@sintef.no. Phone: +47 98283892.

Table of Contents

SUPPORTING INFORMATION 1: Experimental timeline 2

SUPPORTING INFORMATION 2: Literature 3,4-DCA toxicity data 3

SUPPORTING INFORMATION 3: Acute toxicity........ 5

SUPPORTING INFORMATION 4: Hatching timing 6

SUPPORTING INFORMATION 1: Experimental timeline

Table S1.1: Overview of the experiment with age of larvae and timing of sampling for different end points.

**Fish age (dpf)**	**0**	**1**	**2**	**3**	**4**	**5**	**6**	**7**	**8**	**9**	**10**	**11**	**12**	**13**	**14**	**15**
**Date**	**01/04**	**02/04**	**03/04**	**04/04**	**05/04**	**06/04**	**07/04**	**08/04**	**09/04**	**10/04**	**11/04**	**12/04**	**13/04**	**14/04**	**15/04**	**16/04**
**Fish age (d°)**	**6**	**15**	**24**	**33**	**41**	**50**	**58**	**67**	**75**	**83**	**92**	**100**	**109**	**117**	**126**	**135**
Fertilization	x															
Egg arrival		x														
Exposure				(x)	x	x	x	(x)								
Monitoring of survival		x	x	x	x	x	x	x	x	x	X	x	x	x	x	
EHR measurements									x							
Recovery								(x)	x	x	x	x	x	x	x	x
Main hatch												(X)	x	x	(x)	
LHR and morphometry																x
Imaging embryos						x		x	x							

SUPPORTING INFORMATION 2: Literature 3,4-DCA toxicity data

Table S2.1: Toxicity data included in species sensitivity distribution. All data are given in mg/L obtained from 96 h acute toxicity testing on fish where mortality was used as endpoint. References are also given as provided by the EPA Ecotox database (available at https://cfpub.epa.gov/ecotox/search.cfm) and were obtained from peer-reviewed literature.

**Latin name**	**LC50 (mg/L)**	**Source**
Danio rerio	13	1983. Consecutive system of tests for assessment of the effects of chemical agents in the aqutic environment
Danio rerio	8.5	Ensenbach, U., and R. Nagel, 1995. Toxicity of Complex Chemical Mixtures: Acute and Long-Term Effects on Different Life Stages of Zebrafish (Brachydanio rerio), Ecotoxicol.Environ.Saf. 30(2):151-157
Danio rerio	9.8	Ensenbach, U., and R. Nagel, 1995. Toxicity of Complex Chemical Mixtures: Acute and Long-Term Effects on Different Life Stages of Zebrafish (Brachydanio rerio), Ecotoxicol.Environ.Saf. 30(2):151-157
Danio rerio	8.5	Ensenbach, U., and R. Nagel, 1995. Toxicity of Complex Chemical Mixtures: Acute and Long-Term Effects on Different Life Stages of Zebrafish (Brachydanio rerio), Ecotoxicol.Environ.Saf. 30(2):151-157
Danio rerio	9.8	Ensenbach, U., and R. Nagel, 1995. Toxicity of Complex Chemical Mixtures: Acute and Long-Term Effects on Different Life Stages of Zebrafish (Brachydanio rerio), Ecotoxicol.Environ.Saf. 30(2):151-157
Danio rerio	8.59	Zok,S., G. Gorge, W. Kalsch, and R. Nagel. Bioconcentration, Metabolism and Toxicity of Substituted Anilines in the Zebrafish (Brachydanio rerio)
Danio rerio	8.5	Ensenbach, U., and R. Nagel, 1995. Toxicity of Complex Chemical Mixtures: Acute and Long-Term Effects on Different Life Stages of Zebrafish (Brachydanio rerio), Ecotoxicol.Environ.Saf. 30(2):151-157
Gadus morhua	0.635	**This study**
Gobius microps	2.4	Adema,D.M.M., and G.J. Vink. A Comparative Study of the Toxicity of 1,1,2-Trichloroethane, Dieldrin, Pentacholorophenol, and 3,4-Dichloroaniline for Marine and Fresh Water Organisms. Chemosphere10(6): 533-554
Oncorhynchus mykiss	1.94	1988. A comparison of the acute Toxicity of chemicals to fish, rats and mice
Oncorhynchus mykiss	1.94	Hodson,P.V.. A Comparison of the Acute Toxicity of Chemicals to Fish, Rats and Mice
Oncorhynchus mykiss	2.4	Monsanto Co., Acute Toxicity of ACD to Rainbow Trout (Salmo gairdneri)
Oryzias javanicus	32.9	Ibrahim, M. A., et al. "Embryonic toxicity of 3, 4-dichloroaniline (3, 4-DCA) on Javanese medaka (Oryzias javanicus Bleeker, 1854)." Toxicology reports 7 (2020): 1039-1045.
Oryzias latipes	12.9	1992. Biodegradation and Bioaccumulation Data of Existing Chemicals Based on the CSCL Japan
Oryzias latipes	11	Ministry of the Environment, Japan
Perca flavescens	3.1	Schafers, C., and R. Nagel, 1993. Toxicity of 3,4-Dichloroaniline to Perch (Perca fluviatilis) in Acute and Early Life Stage Exposures, Chemosphere 26(9):1641-1651
Perca fluviatilis	3.1	Schafers, C., and R. Nagel, 1993. Toxicity of 3,4-Dichloroaniline to Perch (Perca fluviatilis) in Acute and Early Life Stage Exposures, Chemosphere 26(9):1641-1651
Pimephales promelas	6.99	1987. Toxicity of 3,4-Dichloroaniline to fathead minnows, Pimephales promelas, in acute and early life stage exposure
Pimephales promelas	7.7	1987. Toxicity of 3,4-Dichloroaniline to fathead minnows, Pimephales promelas, in acute and early life stage exposure
Pimephales promelas	8.06	1987. Toxicity of 3,4-Dichloroaniline to fathead minnows, Pimephales promelas, in acute and early life stage exposure
Pimephales promelas	7.55	Broderius,S.J., M.D. Kahl, and M.D. Hoglund. Use of Joint Toxic Response to Define the Primary Mode of Toxic Action for Diverse Industrial Organic Chemicals
Pimephales promelas	9.96	Broderius,S.J., M.D. Kahl, and M.D. Hoglund. Use of Joint Toxic Response to Define the Primary Mode of Toxic Action for Diverse Industrial Organic Chemicals
Pimephales promelas	7	Brooke,L.T., D.J. Call, D.L. Geiger, and C.E. Northcott. Acute Toxicities of Organic Chemicals to Fathead Minnows (Pimephales promelas), Vol. 1
Pimephales promelas	8.06	Brooke,L.T., D.J. Call, D.L. Geiger, and C.E. Northcott. Acute Toxicities of Organic Chemicals to Fathead Minnows (Pimephales promelas), Vol. 1
Pimephales promelas	7.58	Cal DJ, Poirier SH, Knuth ML, Harting SL, Lindberg CA, 1987. Toxicity of 3,4-dichloroaniline to fathead minnows Pimephales promelas, in acute and early life-stage exposures
Pimephales promelas	6.99	Call,D.J., S.H. Poirier, M.L. Knuth, S.L. Harting, and C.A. Lindberg. Toxicity of 3,4-Dichloroaniline to Fathead Minnows, Pimephales promelas, in Acute and Early Life-Stage Exposures

Pimephales promelas	7.7	Geiger, D.L., D.J. Call, and L.T. Brooke, 1988. Acute Toxicities of Organic Chemicals to Fathead Minnows (Pimephales promelas) Volume IV, Ctr.for Lake Superior Environ.Stud., Volume 4, Univ.of Wisconsin-Superior, Superior, WI :355
Pimephales promelas	5.6	Monsanto Co., Acute Toxicity of ACD to Fathead Minnows (Pimephales promelas)
Pimephales promelas	7.58	Russom, C.L., Bradbury, S.P., Broderius, S.J., Drummond, R.A. and Hammermeister, D.E., 1997. PREDICTING MODES OF TOXIC ACTION FROM CHEMICAL STRUCTURE: ACUTE TOXICITY IN THE FATHEAD MINNOW (PIMEPHALES PROMELAS)
Pimephales promelas	7.55	Broderius, S.J., M.D. Kahl, and M.D. Hoglund, 1995. Use of Joint Toxic Response to Define the Primary Mode of Toxic Action for Diverse Industrial Organic Chemicals, Environ.Toxicol.Chem. 14(9):1591-1605 (Author Communication Used)
Pimephales promelas	7	Brooke, L.T., D.J. Call, D.L. Geiger, and C.E. Northcott, 1984. Acute Toxicities of Organic Chemicals to Fathead Minnows (Pimephales promelas), Vol. 1, Center for Lake Superior Environmental Stud., Univ.of Wisconsin-Superior, Superior, WI :414 p.
Pimephales promelas	6.99	Call, D.J., S.H. Poirier, M.L. Knuth, S.L. Harting, and C.A. Lindberg, 1987. Toxicity of 3,4-Dichloroaniline to Fathead Minnows, Pimephales promelas, in Acute and Early Life-Stage Exposures, Bull.Environ.Contam.Toxicol. 38(2):352-358 (OECDG Data File)
Pleuronectes platessa	4.6	Adema,D.M.M., and G.J. Vink. A Comparative Study of the Toxicity of 1,1,2-Trichloroethane, Dieldrin, Pentacholorophenol, and 3,4-Dichloroaniline for Marine and Fresh Water Organisms. Chemosphere10(6): 533-554
Pleuronectes platessa	4.6	Adema,D.M.M., and G.J. Vink. A Comparative Study of the Toxicity of 1,1,2-Trichloroethane, Dieldrin, Pentacholorophenol, and 3,4-Dichloroaniline for Marine and Fresh Water Organisms. Chemosphere10(6): 533-554
Poecilia reticulata	5	Adema,D.M.M., and G.J. Vink. A Comparative Study of the Toxicity of 1,1,2-Trichloroethane, Dieldrin, Pentacholorophenol, and 3,4-Dichloroaniline for Marine and Fresh Water Organisms. Chemosphere10(6): 533-554
Poecilia reticulata	3.5	Adema,D.M.M., and G.J. Vink. A Comparative Study of the Toxicity of 1,1,2-Trichloroethane, Dieldrin, Pentacholorophenol, and 3,4-Dichloroaniline for Marine and Fresh Water Organisms. Chemosphere10(6): 533-554
Poecilia reticulata	8.7	Adema,D.M.M., and G.J. Vink. A Comparative Study of the Toxicity of 1,1,2-Trichloroethane, Dieldrin, Pentacholorophenol, and 3,4-Dichloroaniline for Marine and Fresh Water Organisms. Chemosphere10(6): 533-554
Poecilia reticulata	9	Adema,D.M.M., and G.J. Vink. A Comparative Study of the Toxicity of 1,1,2-Trichloroethane, Dieldrin, Pentacholorophenol, and 3,4-Dichloroaniline for Marine and Fresh Water Organisms. Chemosphere10(6): 533-554
Poecilia reticulata	8.5	1983. Consecutive system of tests for assessment of the effects of chemical agents in the aqutic environment
Poecilia reticulata	9	Adema,D.M.M., and G.J. Vink. A Comparative Study of the Toxicity of 1,1,2-Trichloroethane, Dieldrin, Pentacholorophenol, and 3,4-Dichloroaniline for Marine and Fresh Water Organisms. Chemosphere10(6): 533-554
Poecilia reticulata	8.7	Adema,D.M.M., and G.J. Vink. A Comparative Study of the Toxicity of 1,1,2-Trichloroethane, Dieldrin, Pentacholorophenol, and 3,4-Dichloroaniline for Marine and Fresh Water Organisms. Chemosphere10(6): 533-554
Poecilia reticulata	6.6	Raevsky, O.A., and Dearden, J.C., 2004. Creation of predictive models of aquatic toxicity of environmental pollutants with different mechanisms of action on the basis of molecular similarity and hybot descriptors.
Poecilia reticulata	0.95	Adema,D.M.M., and G.J. Vink. A Comparative Study of the Toxicity of 1,1,2-Trichloroethane, Dieldrin, Pentacholorophenol, and 3,4-Dichloroaniline for Marine and Fresh Water Organisms. Chemosphere10(6): 533-554
Aphanius dispar	0.47	Saeed, S., Al-Naema, N., Butler, J.D. and Febbo, E.J., 2015. Arabian killifish (Aphanius dispar) embryos: A model organism for the risk assessment of the Arabian Gulf coastal waters. Environmental toxicology and chemistry, 34(12), pp.2898-2905.
Aphanius dispar	0.95	Saeed, S., Al-Naema, N., Butler, J.D. and Febbo, E.J., 2015. Arabian killifish (Aphanius dispar) embryos: A model organism for the risk assessment of the Arabian Gulf coastal waters. Environmental toxicology and chemistry, 34(12), pp.2898-2905.
Nothobranchius furzeri	9.75	Philippe, Charlotte, Arnout F. Grégoir, Eli SJ Thoré, Luc Brendonck, Gudrun De Boeck, and Tom Pinceel. "Acute sensitivity of the killifish Nothobranchius furzeri to a combination of temperature and reference toxicants (cadmium, chlorpyrifos and 3, 4-dichloroaniline)." Environmental Science and Pollution Research 25, no. 10 (2018): 10029-10038.
Nothobranchius furzeri	6.61	Philippe, Charlotte, Arnout F. Grégoir, Eli SJ Thoré, Luc Brendonck, Gudrun De Boeck, and Tom Pinceel. "Acute sensitivity of the killifish Nothobranchius furzeri to a combination of temperature and reference toxicants (cadmium, chlorpyrifos and 3, 4-dichloroaniline)." Environmental Science and Pollution Research 25, no. 10 (2018): 10029-10038.

SUPPORTING INFORMATION 3: Acute toxicity

Table S3.1: LC_{50} values (with 95% confidence intervals) for Atlantic cod exposed to 3,4-DCA estimated at different time points during exposure (5–7 dpf) and during recovery (8–14 dpf) by using sigmoidal dose-responses (variable slope).

**Time (dpf)**	**LC_{50} (µg/L) with 95% confidence intervals**
5 dpf	1781 (979.7-3237)
6 dpf	773.3 (714.1-837.3)
7 dpf	635.1 (589.0-684.8)
8 dpf	599.3 (551.4-651.4)
9 dpf	587.4 (534.3-645.8)
10 dpf	570.8 (517.9-629.1)
11 dpf	527.0 (472.8-587.3)
12 dpf	487.3 (429.8-552.5)
13 dpf	433.4 (360.3-521.4)
14 dpf	310.3 (275.0-350.0)

SUPPORTING INFORMATION 4: Hatching timing

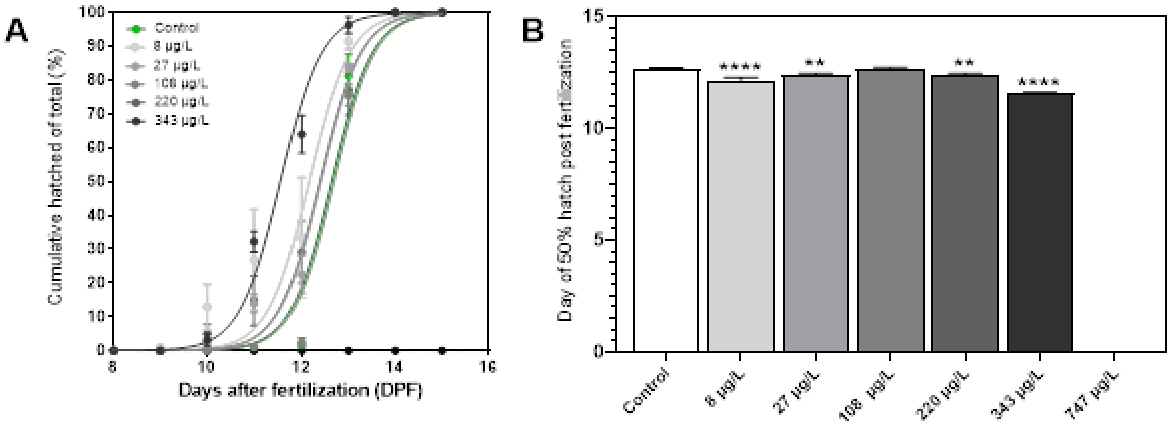

Figure S4.1: A: Cumulative hatching in % of total hatched egg as a function of days post fertilization (DPF) for cod embryos exposed to different concentrations of 3,4-DCA. Values are given as mean ± SD (N=3). B: Calculated day post hatch 50% of embryos were hatched. Significant differences are denoted **p<0.01 and ***p<0.0001.
